# Magnetically Guided Catheters, Micro- and Nanorobots for Spinal Cord Stimulation

**DOI:** 10.3389/fnbot.2021.749024

**Published:** 2021-10-20

**Authors:** Harun Torlakcik, Can Sarica, Patrick Bayer, Kazuaki Yamamoto, Christian Iorio-Morin, Mojgan Hodaie, Suneil K. Kalia, Joseph S. Neimat, Juha Hernesniemi, Anuj Bhatia, Bradley J. Nelson, Salvador Pané, Andres M. Lozano, Ajmal Zemmar

**Affiliations:** ^1^Department of Neurosurgery, Henan Provincial People's Hospital, Henan University People's Hospital, Henan University School of Medicine, Zhengzhou, China; ^2^Multi-Scale Robotics Laboratory, Swiss Federal Institute of Technology (ETH) Zurich, Zurich, Switzerland; ^3^Division of Neurosurgery, Department of Surgery, University of Toronto, Toronto, ON, Canada; ^4^Faculty of Medicine, Ludwig Maximilians University Munich, Munich, Germany; ^5^Department of Neurosurgery, University of Sherbrooke, Sherbrooke, QC, Canada; ^6^Department of Neurosurgery, School of Medicine, University of Louisville, Louisville, KY, United States; ^7^Department of Anesthesia and Pain Medicine, University Health Network, University of Toronto, Toronto, ON, Canada

**Keywords:** nanorobot, spinal cord stimulation, magnetic steering, neurorobotics, microrobot

## Abstract

Spinal cord stimulation (SCS) is an established treatment for refractory pain syndromes and has recently been applied to improve locomotion. Several technical challenges are faced by surgeons during SCS lead implantation, particularly in the confined dorsal epidural spaces in patients with spinal degenerative disease, scarring and while targeting challenging structures such as the dorsal root ganglion. Magnetic navigation systems (MNS) represent a novel technology that uses externally placed magnets to precisely steer tethered and untethered devices. This innovation offers several benefits for SCS electrode placement, including enhanced navigation control during tip placement, and the ability to position and reposition the lead in an outpatient setting. Here, we describe the challenges of SCS implant surgery and how MNS can be used to overcome these hurdles. In addition to tethered electrode steering, we discuss the navigation of untethered micro- and nanorobots for wireless and remote neuromodulation. The use of these small-scale devices can potentially change the current standard of practice by omitting the need for electrode and pulse generator implantation or replacement. Open questions include whether small-scale robots can generate an electrical field sufficient to activate neuronal tissue, as well as testing precise navigation, placement, anchoring, and biodegradation of micro- and nanorobots in the *in vivo* environment.

## Introduction

Spinal cord stimulation (SCS) is a well-described treatment for medically refractory pain (Grider et al., 2016). SCS has recently also gained interest for improving locomotion (Pinto de Souza et al., 2017; Rohani et al., 2017; Wagner et al., 2018; Courtine and Sofroniew, 2019; Goudman et al., 2020; Prasad et al., 2020). However, SCS implantation can be associated with a relatively high injury rate, ranking spinal cord stimulator placement as the third-highest leading cause of injury among all medical devices after metal hip prostheses and insulin pumps (Taccola et al., 2020). Magnetic navigation systems (MNS) are an emerging technology permitting precise and dynamic steering of surgical probes (Zemmar et al., 2020). Magnets placed external to the patient's body are used to guide a surgical probe equipped with a magnetic tip. They have been successfully applied in endovascular cardiovascular interventions (Ali et al., 2016). To date, neurosurgical applications of MNS have been limited to preclinical studies (Hong et al., 2019, 2021), while spinal applications remain unexplored. For SCS, this technology harbors several benefits, including (i) enhanced flexibility to navigate the SCS electrode to the target location during its placement, (ii) reduced procedure time and cost in the operating room, and (iii) non-invasive ability to adjust the SCS electrode post-operatively. In addition to steering tethered probes, MNS can also be exploited for the manipulation of smaller and less invasive untethered devices, such as magnetically actuated micro- and nanorobots (Nelson et al., 2010; Dulińska-Litewka et al., 2019; Hwang et al., 2020; Soto et al., 2020; Wang et al., 2021), which could take full advantage of the magnetically driven deformational change and piezoelectric properties (Wang et al., 2010; Ciofani and Menciassi, 2012; Chen et al., 2015, 2017a,b, 2018, 2019; Rajabi et al., 2015; Ribeiro et al., 2016; Hoop et al., 2017; Mei et al., 2020) that can occur at that scale. They may therefore represent a potent alternative to traditional neuromodulation with the advantage that they could be placed with less invasive procedures and would obviate the need for placement and replacement of pulse generators (Christiansen et al., 2019).

The goal of this perspective article is to review conventional SCS implantation techniques together with their related complications and limitations, and to reflect on how magnetically steered leads and untethered micro- and nanorobots could be implemented to improve the existing standard.

## Spinal Cord Stimulation: Current Technique, Limitations and Complications

**Figure 1:**
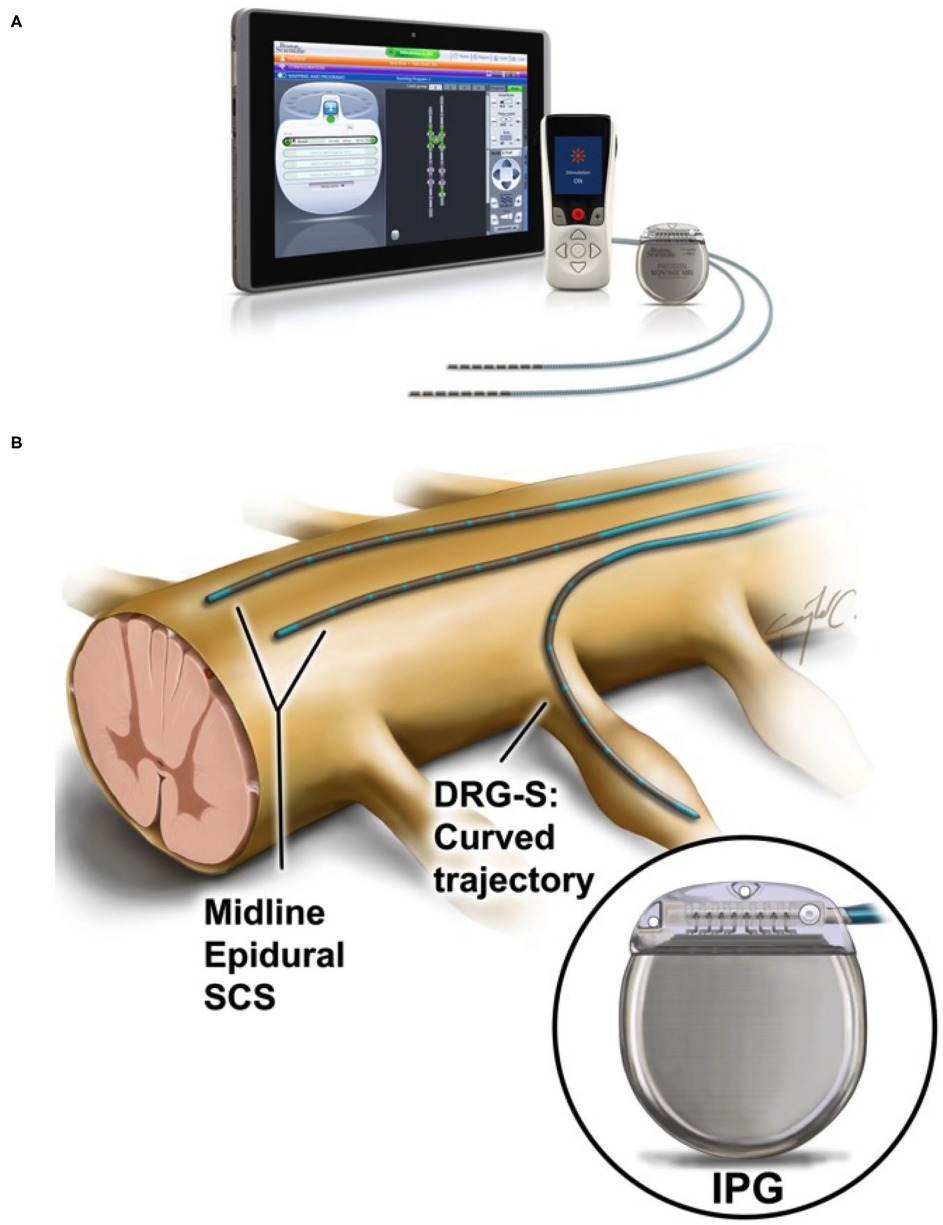
**(A)** Overview of a spinal cord stimulation system. The components consist of lead electrodes, the implantable pulse generator (IPG), a remote control for the patient and a tablet for the physician to program the device after implantation. Adapted with permission from Boston Scientific. **(B)** Midline electrode placement in the epidural space for spinal cord stimulation and dorsal root ganglion stimulation (DRG-S). Note the difference in trajectory curvature between these two applications. In both instances, the trajectory path depends on obstacles (e.g., blood vessels) and confinement created by spinal degenerative disease, etc. Reproduced with the permission of Cura Canaz Medical Arts.

The traditional SCS system includes the SCS lead(s), the implantable pulse generator (IPG), and the extension wire(s), which connect(s) the lead(s) to the IPG (Figure 1A). SCS implantation is typically divided into two stages. An initial trial stage assesses stimulation efficacy, followed by implantation of an IPG in patients for whom stimulation is effective during the trial. The lead implantation can be done either by laminectomy (open surgery) or *via* a less invasive (percutaneous) technique, which allows for the placement of smaller electrodes. For the latter, in our experience, manual control of the lead can be challenging, especially in presence of scar tissue, and may be associated with prolonged operating room (OR) time, patient discomfort, increased cost, and potential complications. This maneuver can be impeded with complex anatomy, often seen in patients with degenerative spine disease, or for targets in which the SCS lead has to be placed within confined spaces such as the dorsal root ganglion (Caylor et al., 2019) (Figure 1B). After initial lead placement, the clinical effect of SCS is tested. The criteria of success for the trial stage are typically determined by reduction in pain scores of 50% or more relative to the baseline. Candidates who have satisfactory results during the trial stage undergo permanent implantation of an implantable pulse generator (IPG), which is placed subcutaneously or subfascial in the gluteal or abdominal region (Rock et al., 2019). In some cases, a repeat surgery to adjust the electrode lead(s) is needed to determine ideal lead placement.

The most common complications of SCS implantation include electrode migration, hardware malfunction and fracture of electrodes, tolerance to SCS, infection, cerebrospinal fluid (CSF) leakage and pain or hematoma/seroma at the pulse generator site (Bendersky and Yampolsky, 2014). Electrode migration occurs in 13–22% of SCS patients (Taccola et al., 2020), is more common with the less invasive percutaneous technique, and has been reported as the most frequent reason for repeat surgery (Turner et al., 2004), resulting in increased risk for the patient and additional operative time and cost. Even with the lowest reported incidence of 2% (Gazelka et al., 2015), when considering the high volume of SCS procedures worldwide, this percentage still corresponds to a considerably high number of patients. Tolerance to SCS usually develops after 1 year in around 10–29% of patients and often requires repeat surgery with alteration of the tip location (Taccola et al., 2020). Finally, infection related to SCS occurs in ~5% of the cases (Bendersky and Yampolsky, 2014). Notably, increased operating time is a known risk factor for infection and revision surgeries trend toward longer operating times and thus higher infection rates.

## Benefits of Magnetic Navigation Systems for SCS Lead Placement

**Figure 2:**
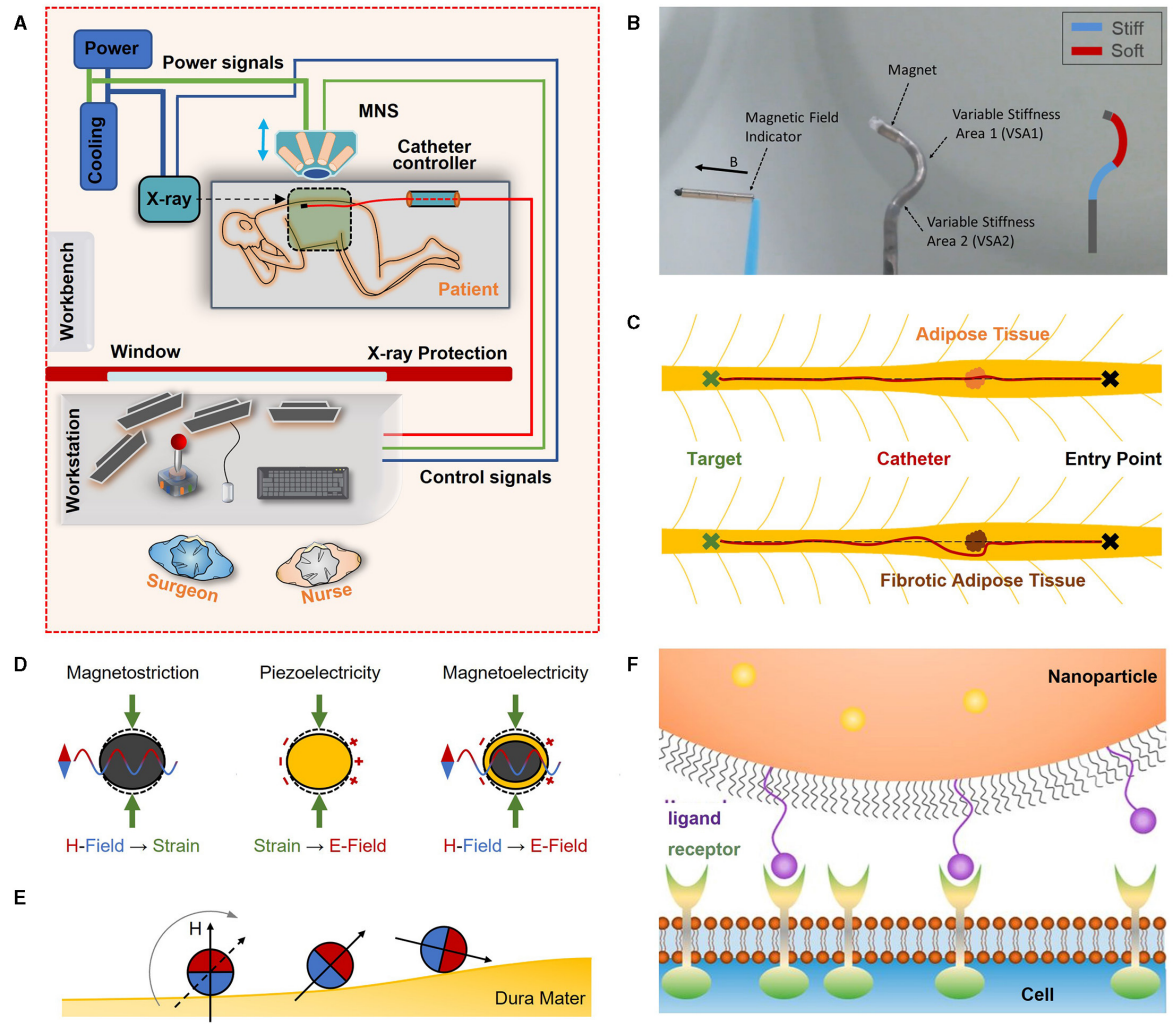
**(A)** Envisioned outpatient setting for remote SCS lead placement with MNS. Lead tip angle and direction guided by magnetic fields and advanced in the epidural space with a catheter controller (adapted with permission from Zemmar et al., 2020). **(B)** Variable stiffness catheters enabling complex catheter shapes (adapted with permission from Chautems et al., 2017). **(C)** Forces contributing to trajectory deflection. Under ideal conditions and tissue composition in the epidural space, with enough force, the catheter can be guided through adipose tissue to follow the mid-line (dashed line) from entry point to the target location. When the adipose tissue is fibrotic and too dense, the catheter can be deflected, and the surgeon must adjust the tip to redirect the catheter to the desired path. **(D)** Operation principle of magnetostrictive, piezoelectric and magnetoelectric composite core-shell materials. Magnetostrictive materials can show deformation when exposed to magnetic fields due to internal mechanical strain generation. When piezoelectric materials are exposed to mechanical strain and deformation, electrical surface charges can be generated. Both effects can be combined in a magnetoelectric composite material: A magnetostrictive core is deformed when in presence of a magnetic field. This deformation is transferred to a bonded piezoelectric shell, which in turn generates electrical surface charges. **(E)** Alignment of magnetic moment of microrobots with an external magnetic field. Rotation of the external magnetic field will result in a rolling motion of the microrobot on the dura mater. Due to friction between microrobot and dura mater, the microrobot will advance in the epidural space tumbling over the surface and approaching the target location. **(F)** Particle targeting. Surfaces of particles can be decorated with ligands which are designed to bond to receptors on the cell surface to immobilize particles at the desired target location (adapted with permission from Scheepers et al., 2020).

The use of MNS offers several potential benefits for SCS lead implantation, including precise navigation control during SCS lead placement, the ability for non-invasive post-operative re-adjustment of the SCS lead(s), decreased OR time and cost, reduced radiation exposure to the surgeon and patient and, in the light of the current Covid-19 pandemic, reduced direct contact with the patient and therefore a decreased risk for pathogen mitigation (Figure 2A) (Zemmar et al., 2020).

### Increased Navigation Control and Accuracy During SCS Lead Placement

Magnetic steering provides improved dexterity, precision, and safety of implantation over manual steering. Magnetic forces and torques are used in MNS to control the tip angle and steer the lead in the desired direction. In a recent study, Hong et al. (2021) demonstrated precise following of pre-drawn trajectories with a radius as small as 30 mm (Petruska et al., 2016) when using an MNS compared to manual steering in a brain phantom and an *ex-vivo* pig brain. Such radii extend the reachable workspace and allow for greater accuracy (Ilami et al., 2020). In a brain model intended to simulate deep brain stimulation of the subthalamic nucleus, electrode positioning with a precision of 1.16–1.29 mm was achieved with MNS (Hong et al., 2021). The use of MNS allows correction of the trajectory in real time during advancement of the electrode and offers the surgeon a yet unidentified degree of flexibility to adjust the surgical probe to follow the desired trajectory. This facilitates accurate maneuvering along complex paths while minimizing tissue damage (Hong et al., 2019, 2021; Ilami et al., 2020). MNS also allow for steering control at longer distances, e.g., the lead could be inserted at the thoraco-lumbar level (which is safer than more cranial insertion points) and guided to the cervical level, or a combined cervical and thoracic lead could be guided through a single entry and navigated to both targets. With the current standard, the insertion is usually closer to the target location to facilitate navigation control as dexterity of the probe's tip decreases the further away the target is located. Lead manipulation is optimized when MNS are combined with variable stiffness catheters that consist of multiple segments with independent stiffness control (Figure 2B) (Chautems et al., 2017). This technology can be applied to SCS leads to permit flexibility for the surgeon during lead placement. Paired with force feedback, the surgeon's armamentarium can be equipped with another degree of safety to minimize injury of critical structures, i.e., whenever the catheter approximates a critical structure, the surgeon receives feedback to avoid the respective structure.

### Re-adjustment of the SCS Lead

For the minimal-invasive percutaneous trial, SCS placement is usually done when the patient is awake in order to receive patient feedback in response to stimulation when determining the optimal lead location. This process could be time-consuming until optimal lead placement is achieved and it creates positional discomfort for the patients since they are awake and in the prone position. An advantage of MNS would be that SCS leads could be placed outside an operating room (similar to a lumbar puncture, which can be done in a physician's office) through a Touhy needle into the epidural space. After placing the SCS lead in the epidural space, the MNS could be utilized to navigate the SCS electrode to the desired location. A pre-operative magnetic resonance imaging (MRI) could assist in plotting a pre-defined trajectory for the MNS. Verification of the location of the SCS electrode with fluoroscopy can be obtained if desired. Verbal feedback can be given by the patient regarding the location of the induced paresthesia while being awake and in a comfortable position. This setting would enhance the comfort for the patient and the surgeon. Another major benefit of MNS is the possibility to re-adjust the SCS electrodes in an outpatient setting without the necessity of a repeated surgical procedure. This may be necessary if the lead migrates or the analgesic benefit is lost, in suboptimal postoperative coverage with the evolution of the pain syndrome to new body regions, or for testing new stimulation waveforms over time, e.g., conventional stimulation vs. burst stimulation vs. new algorithms such as Differential Target Multiplexed^TM^ SCS (Vallejo et al., 2020).

### Minimizing Radiation Exposure and Infectious Risk

The percutaneous lead placement technique necessitates fluoroscopic guidance during needle insertion and lead placement phases. MNS can reduce radiation exposure of healthcare workers (Yuan et al., 2017) as the procedure can be performed remotely, i.e., the surgeon is located outside the surgical suite behind a radiation barrier (Figure 2A). Additionally, remote and contactless surgery reduces the risk of pathogen spread among healthcare workers and patients by decreasing direct contact, which is a desired effect in the light of the current Covid-19 pandemic (Zemmar et al., 2020).

## Technical Considerations for Magnetic Navigation of Tethered Surgical Probes

### Major Differences Between Cortical and Spinal Epidural Spaces

In contrast to navigating a probe through cortical tissue, the spinal epidural space is a relatively “empty” cavity with connective adipose tissue and blood vessels (Newell, 1999; Grady et al., 2000). The challenges from an engineering point of view in positioning and aligning a magnetic stimulation lead are different from the challenges that surgeons face when they must accurately guide and position a flexible magnetic needle. Surrounding tissue supports the catheter body and prevents free movement of tip-distant sections, allowing precise path selection and navigation (Petruska et al., 2016). However, when navigating within the spinal epidural space, support from surrounding tissue does not exist and the catheter body can move relatively freely within the space. Hence, buckling and alignment of the tip are major problems that have to be considered, and methods need to be developed to steer the magnetic SCS lead precisely despite unpredictable anatomical obstacles. In current procedures, connective adipose tissue (e.g., fibrous septa in the dorsal epidural space) is often penetrated, and the catheter is guided straight through whenever the composition of the adipose tissue permits. When the adipose tissue is fibrotic, the catheter will simply evade and be re-directed due to tissue forces, and action from the surgeon or navigation system must be taken to redirect the catheter to the desired path (Figure 2C). Another critical factor is the steering radius, as the diameter of the spinal canal requires more precise turning compared to cortical navigation. While 30 mm radii have been precisely followed in the brain (Petruska et al., 2016), the steering radius of the tip in SCS is closer to 15 mm, which has to be tested in *in vivo* environments.

### Design of the Magnetic Stimulation Lead

Sufficient flexibility of the catheter tip is necessary to provide steering freedom and after removal of the magnetic field, “stress-relaxation” (micro-shattering of the electrode tip) should be avoided (Jonathan and Groen, 2005; Petruska et al., 2016). Stimulation leads for the epidural space are less flexible as they have to be more resistant to buckling. In general, when designing a magnetic catheter, one is faced with the challenge of catheter flexibility and the necessary amount of a magnetic volume to bend the tip and catheter rigidity for prevention of buckling during catheter advancement (Carey et al., 2006; Chautems et al., 2020). This optimization problem is particularly difficult in the epidural space, as connective tissue, open cavities, and long insertion depths generally require a stiff catheter design. On the other hand, increased steering freedom, would grant access to locations that are difficult to reach, such as dorsal root ganglia (Jonathan and Groen, 2005; Swaney et al., 2013; Caylor et al., 2019). A variable stiffness catheter design could be a solution to this optimization problem (Figure 2B). Alternatively, a simple variable stiffness catheter design can be based on current technology, where the stiffness of the stimulation lead is controlled by the degree of insertion of the guidewire (Schulder, 2003). The integrated magnets in the tip must be arranged and designed to comply with current surgical access methods through Tuohy needles. A neodymium-iron-boron (NdFeB) permanent tip has been suggested with magnetically guided catheters, as NdFeB has the highest magnetic remanence among commercially available magnets (Gutfleisch et al., 2011; Hong et al., 2021). However, these magnets are not biocompatible and must be shielded from the body to avoid inflammatory reactions by means of biocompatible enclosures with Parylene (Evans and McDonald, 1995; Prodromakis et al., 2009). Additionally, optic fibers with fiber-Bragg-gratings sensors (FBGs) that reflect light at a wavelength that is proportional to mechanical (and thermal) strain on the fiber can be incorporated into the magnetic tip for contact-force measurements (Di Natali et al., 2016; Khan et al., 2019).

### Challenges of Magnetically Guided Stimulation Leads

Strong lateral tissue forces may require relatively high magnetic field strengths to hold the electrode tip on the pre-planned trajectory. Alignment could be achieved by magnetic fields in the range of 50–100 mT. Deviations from the trajectory could either be corrected by turning and angling the magnetic tip in the direction of the trajectory or by the application of lateral gradient fields to counter lateral tissue forces directly if they are low magnitude. Obstacles such as connective tissue might increase the deviation from the trajectory, which the control algorithm has to smartly consider. For improved detection of these obstacles, surgeon and control algorithms would require 3-dimensional visual feedback using biplanar fluoroscopic imaging during electrode guiding and placement, or in combination with other imaging techniques, such as pre-operative MRI and trajectory modeling (Jonathan and Groen, 2005; Hong et al., 2015, 2019; Hu et al., 2018). Additional feedback and situational awareness could be delivered by FBGs (Su et al., 2017). For semi-automated procedures, haptic feedback devices could be linked with FBGs signals to provide a sense of touch to the surgeons' hand (El Rassi and El Rassi, 2020). When the lead has been successfully navigated to the target location, surgeons can proceed as usual and connect an IPG manually. Alternatively, systems could be developed, in which the trial IPG is directly integrated in the catheter advancer unit with semi-automated paresthesia mapping. The remaining surgery would proceed in the traditional sense. In case of electrode migration, magnetic forces created by magnetic gradients perpendicular or parallel to the aligned field could be used to non-invasively and precisely move the electrode to the desired stimulation site. However, this motion will be limited as the length of the catheter cannot be extended and frictional forces should not be too large, which will have to be verified in *in vivo* trials.

## Utilizing Untethered Magnetic Micro- and Nanorobots for SCS

Magnetically controlled probes could be the precursor of untethered magnetic devices. Micro- or nanorobots are small-scale devices designed to perform minimally-invasive interventions and are powered by external power sources (Colberg et al., 2014; Zeeshan et al., 2014; Rao et al., 2015; Chen et al., 2017a; Soto et al., 2021). To be considered for neurostimulation, micro- and nanorobots must be able to generate an electrical field. Since neuronal stimulation in the brain has already been demonstrated (Yue et al., 2012; McGlynn et al., 2020; Singer et al., 2020; Kozielski et al., 2021), this can serve as an intriguing technology for SCS. In micro- and nanorobotic applications, magnetoelectric devices are mostly made from magnetoelectric composites that exhibit coupling between ferromagnetism and ferroelectricity (Spaldin and Fiebig, 2005; Wang et al., 2010) and consist of a structural combination of magnetostrictive and piezoelectric materials (Figure 2D) (Wang et al., 2010). Micro- and nanodevices have the potential for remote neurostimulation without requiring a stimulation lead and implantation or replacement of an IPG, which saves additional surgeries for the patient (Nan et al., 2008; Armin et al., 2012). An external field generator can be attached to the skin and used to create deeply penetrating magnetic fields and generate electric charges non-invasively and remotely. By modulation of the external magnetic field input, the electrical field amplitude and shape could be adjusted as desired (Nan et al., 2008; Armin et al., 2012). An important question is if and how the induced fields will benefit the patient as applied stimuli are not electric currents, as is the case with current SCS technology (Dones and Levi, 2018). However, early results in deep brain stimulation with multiferroics demonstrate a beneficial effect, which is promising for application in the spinal cord (Singer et al., 2020; Kozielski et al., 2021). Future studies are needed to directly measure neuronal activation by piezoelectric and magnetostrrictive properties.

Using external magnetic fields, micro- and nanorobots can be propelled with magnetic gradient forces or magnetic torque through rotating fields (Pawashe et al., 2009; Chen et al., 2018). In the epidural space with minute amounts of biological fluids, micro- and nanorobot delivery will be complicated by surface friction and adhesion forces. Drag forces, that are often a limiting cause for targeted micro- and nanorobot delivery, may not need to be considered in the epidural space, as little flow is present. The use of rotating fields may manage these challenges as micro- or nanorobots could overcome boundary forces while rolling on the surface (Figure 2E) or exhibit a “surface walker” locomotion behavior (Peyer et al., 2013).

A critical factor that requires further investigation lies in the permanent attachment of the micro- and nanorobots on the dura mater through chemical modification with targeting ligands (Figure 2F), peptides or antibodies to avoid migration from stimulation site (Cheng et al., 2015; Tietjen et al., 2018; Scheepers et al., 2020) and offer regular neurostimulation for the patient. The choice of composite materials must be carefully considered as they need to fulfill all safety criteria for permanently implantable devices (Soto et al., 2020, 2021). Although a few biocompatible magnetostrictive and piezoelectric materials exist (Wang et al., 2010; Rajabi et al., 2015; Ribeiro et al., 2018), the compatibility of these materials in the spinal epidural space or subarachnoid space needs to be investigated in future research as literature suggests that different tissue types show different cellular responses (Dulińska-Litewka et al., 2019).

## Conclusion

Magnetic navigation of tethered probes represents a novel technology that offers potential to improve dexterity control, the option for postoperative readjustment of the electrode to modulate the volume of activated tissue, increases safety, and reduces cost in spinal cord stimulation surgery. Future steps include testing and integration of tethered probes in clinical environments. Untethered micro- and nanorobots represent an innovative future perspective, for which the effect of the generated electric field has to be tested, as well as how this can activate neuronal tissue. Navigation, release, anchoring, and biocompatibility of these small-scale devices are further open challenges that require proof-of-concept studies and *in vivo* verification.

## Data Availability Statement

The original contributions presented in the study are included in the article/supplementary material, further inquiries can be directed to the corresponding author/s.

## Author Contributions

HT, CS, PB, and JN wrote the manuscript and designed research. MH and AB edited the manuscript. SK and JH wrote the manuscript. BN, SP, AL, and AZ wrote the manuscript, conceptualized, and designed research. All authors contributed to the article and approved the submitted version.

## Funding

This work was supported by grants from the Heidi Demetriades Foundation, the ETH Zurich Foundation, and the Henan Provincial People's Hospital Outstanding Talents Founding Grant Project to AZ. SP acknowledges the European Research Council (ERC) under the European Union's Horizon 2020 research and innovation programme (Grant agreement No. 771565).

## Conflict of Interest

The authors declare that the research was conducted in the absence of any commercial or financial relationships that could be construed as a potential conflict of interest.

## Publisher's Note

All claims expressed in this article are solely those of the authors and do not necessarily represent those of their affiliated organizations, or those of the publisher, the editors and the reviewers. Any product that may be evaluated in this article, or claim that may be made by its manufacturer, is not guaranteed or endorsed by the publisher.
